# A new open access journal for a rapidly evolving biomedical field: introducing *Molecular Cytogenetics*

**DOI:** 10.1186/1755-8166-1-1

**Published:** 2008-03-26

**Authors:** Yuri B Yurov, Thomas Liehr, Lisa G Shaffer, Ivan Y Iourov, Svetlana G Vorsanova

**Affiliations:** 1 National Research Center of Mental Health, Russian Academy of Medical Sciences, Moscow 119152, Russia; 2 Institute of Human Genetics and Anthropology, Jena 07740, Germany; 3 Signature Genomic Laboratories, Spokane, WA 99202, USA; 4 Institute of Pediatrics and Children Surgery, Rosmedtechnologii, Moscow, 127412, Russia

## 

*A method is more important than a discovery, since the right method will lead to new and even more important discoveries*.

-*Lev D. Landau, Nobel Prize laureate*

Welcome to *Molecular Cytogenetics*. We are proud to introduce you to a new home for biomedical research focused on all aspects of chromosome biology and applications of molecular cytogenetic techniques in all areas of biomedicine. *Molecular Cytogenetics *[1] represents the first open access source for research concerning molecular cytogenetic techniques. *Molecular Cytogenetics *will be a valuable resource for researchers all over the world, both those who are already experts and those entering the field.

Molecular cytogenetics comprises a set of the techniques operating with either the entire genome or with specific DNA sequences to study genome structure and functions at the chromosomal level. Molecular cytogenetics can also be defined as a specific focus of biomedical sciences targeted at studying chromosomes at molecular and single-cell resolutions and at all stages of the cell cycle. Furthermore, the area of molecular cytogenetic technique applications has come to encompass almost everything from applied research in clinical and cancer genetics to basic studies in cellular and structural biology, genomics, genetics, and neurosciences. Thus, a journal focused on molecular cytogenetics is not limited to technical issues but will also address advances in current biomedical research. *Molecular Cytogenetics *will provide multilateral coverage of numerous applied and basic aspects of current biomedicine.

Taking into account the growing amount of biomedical journals available, one can argue whether we need another one. However, any scientist working on theoretical and practical aspects of chromosome biology would undoubtedly agree that there are few journals covering this area of biology, and that there are no journals providing immediate open access. We performed an informal analysis (Figure 1) that shows the consistent interest of researchers to publish their original molecular cytogenetic data in peer-reviewed journals. However, to read the largest proportion of articles (over 70%) related to molecular cytogenetics, one has to have a subscription. Therefore, numerous scientists all over the world, especially those in the developing countries, miss the opportunity to keep abreast with the latest advances in molecular cytogenetics. A number of journals indirectly related to molecular cytogenetics make articles free toaccess after a set period of time, but still non-subscribers, regardless of their intellectual potential, have to operate with backward data, therefore having fewer opportunities to take a well deserved place in competition with those who have immediate access. Moreover, a recent survey describes open access articles as benefiting science by accelerating dissemination and uptake of research findings [2]. Since there are no open access journals in the fields of chromosome biology and molecular cytogenetics, the present journal fills this gap in current biomedical publishing.

**Figure 1:**
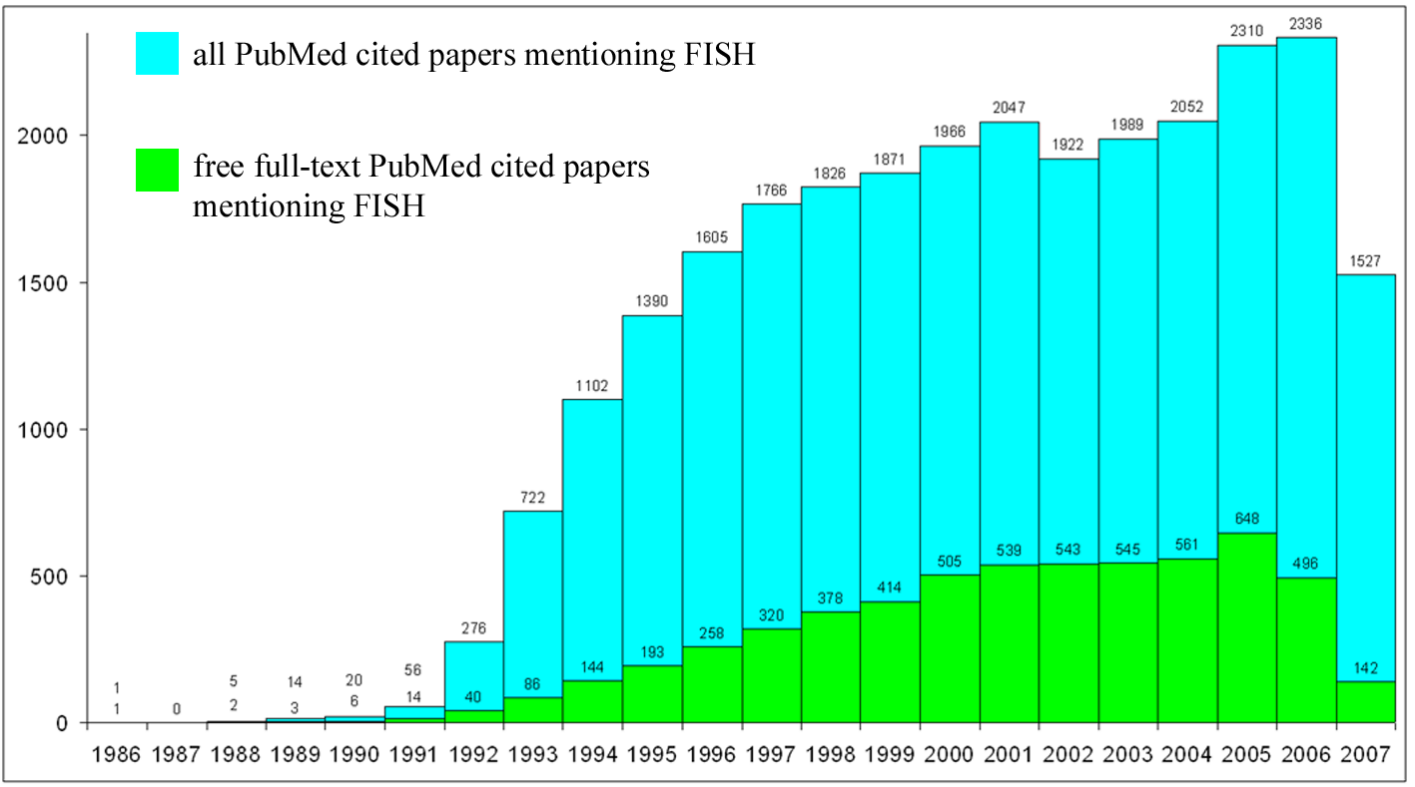
Number of citations in the PubMed database for the keywords "fluorescence *in situ *hybridization" (FISH), one of the most applied molecular cytogenetic techniques, by publication year. Blue bars represent the number of papers mentioning FISH; green bars represent the number of papers mentioning FISH that have links to the free full-text on the publisher's site.

Molecular cytogenetics is a rapidly evolving field of biomedicine. Throughout the last decade, significant breakthroughs in molecular cytogenetics have been made. This can be attributed in part to the establishment of new composite molecular cytogenetic technologies that have significantly enhanced diagnosis in medical and cancer genetics, for example the conception of molecular karyotyping using microarray (array-CGH) and more sophisticated digital karyotyping for identification of subtle genomic imbalances and copy number changes at molecular resolutions. New technologies are expected to follow at this rapid pace. Molecular cytogenetic techniques possess the potential not only to become more efficient through further enhancements and modifications, but also to lead to new and important discoveries in biomedicine. In this context, the lack of a journal exclusively dedicated to molecular cytogenetics can be considered an omission. Therefore, *Molecular Cytogenetics *fills another gap in current biomedical publishing.

*Molecular Cytogenetics *aims to become a significant international participant in the fields of genetics and cell biology. The purpose of the journal is, therefore, to publish timely and high-quality articles concerning chromosome biology and the application of molecular cytogenetic techniques in all areas of biomedicine. To achieve this, the Editorial Board of *Molecular Cytogenetics *gathers well-known experts from all over the world [3]. To make a decision whether a manuscript fits the format of publishing in *Molecular Cytogenetics*, the Editors-in-Chief or corresponding specialists from the Editorial Board will immediately screen a submission and appropriate manuscripts will be sent for peer review to two independent reviewers. Based on their reports and scoring, a final decision will be made regarding acceptance of the manuscripts. Editors-in-Chief reserve the right to make final decisions concerning each manuscript submitted to the journal. At first sight our peer review system does not significantly differ from that proposed by other journals. However, we encourage our Editorial Board members as well as reviewers to be motivated critical advisers for improving a manuscript rather than rigorous censors for biomedical publishing, which is unfortunately a common experience for anyone who has ever tried to publish his or her original research results.

*Molecular Cytogenetics *is published by the open access publisher, BioMed Central [4]. BioMed Central ensures all articles published in the journal are listed in PubMed and archived in PubMed Central, and other national archives. When submitting an article authors will be required to complete a declaration of competing interests. In addition, for all articles accepted for publication an article-processing charge will be levied, to cover the costs incurred by open access publication. We believe that authors' ability to publish research should be governed only by the quality of the work and not financial limitations. Therefore, article-processing charges (APCs) will be comparable to and no more cost-prohibitive than the color figure publication fees assessed by most scientific journals. In addition, over 300 institutes have committed to a BioMed Central institutional membership[5], whereby they cover the full or partial cost of the APC, and a number of funding bodies actively support open access and explicitly allow direct use of their grants to cover APCs [6]. Waivers can be requested by authors who have a genuine inability to pay the charge. The most common reason for publication rejection being the lack of space in a journal, another advantage of *Molecular Cytogenetics *is that there are no limitations regarding space or color figures.

Considering all the advantages *Molecular Cytogenetics *possesses, we believe the journal to be of great benefit to the field of biomedicine. We invite you to work together with us to create this new and high-visibility open-access forum for the biomedical sciences.

## Competing interests

YBY, TL, and LGS are the Editors-in-Chiefs of *Molecular Cytogenetics*, IYI is the managing editor, and SGV is a member of the Editorial board. The authors declare that they have no competing interests.
